# A brief tool to assess capacity to consent for medical care among homeless individuals with problematic substance use: study protocol

**DOI:** 10.1186/0778-7367-71-11

**Published:** 2013-05-08

**Authors:** Darlene Taylor, Louise Masse, Anita Ho, Michael L Rekart, Mark Tyndall, Bonnie Henry, Joanne Clifton, Laurenna Peters, Gina Ogilvie, Jane Buxton

**Affiliations:** 1 BC Centre for Disease Control, University of British Columbia, 655 West 12th Avenue, Vancouver, BC, V5Z 4R4, Canada; 2 Department of Pediatrics/School of Population and Public Health, University of British Columbia, 4480 Oak Street, L408, Vancouver, BC, V6H 3V4, Canada; 3 Centre for Applied Ethics, University of British Columbia, 6356 Agricultural Road, Vancouver, BC, V6T 1Z2, Canada; 4 School of Population and Public Health, University of British Columbia, 2206 East Mall, Vancouver, BC, V6T 1Z3, Canada; 5 Department of Infectious Diseases, University of Ottawa, 75 Laurier Avenue East, Ottawa, ON, K1N 6N5, Canada; 6 Department of Surgery, University of British Columbia, 899 West 12th Avenue, Vancouver, BC, V5Z 1M9, Canada

**Keywords:** Capacity to consent, Substance use, Psychometric instruments, Vulnerable populations

## Abstract

**Background:**

Public health care increasingly uses outreach models to engage individuals who are marginalized, many of whom misuse substances. Problematic substance use, together with marginalization from the health care system, among homeless adults makes it difficult to assess their capacity to consent to medical care. Tools have been developed to assess capacity to consent; however, these tools are lengthy and unsuitable for outreach settings. The primary objective of this study is to develop, validate, and pilot a brief but sensitive screening instrument which can be used to guide clinicians in assessing capacity to consent in outreach settings. The goal of this paper is to outline the protocol for the development of such a tool.

**Methods/Design:**

A brief assessment tool will be developed and compared to the MacArthur Competency Assessment Tool for Treatment (MacCAT-T). As list of 36 possible questions will be created by using qualitative data from clinician interviews, as well as concepts from the literature. This list will be rated by content experts according to the extent that it corresponds to the test objectives. The instrument will be validated with 300 homeless adult volunteers who self-report problematic substance use. Participants will be assessed for capacity using the MacCAT-T and the new instrument. A combination of Classical Test Theory and advanced psychometric methods will be used for the psychometric analysis. Corrected Item-Total correlation will be examined to identify items that discriminate poorly. Guided exploratory factor analysis will be conducted on the final selection of items to confirm the assumptions for a unidimensional polytomous Rasch model. If unidimensionality is confirmed, an unstandardized Cronbach Alpha will be calculated. If multi-dimensionality is detected, a multidimensional Rasch analysis will be conducted. Results from the new instrument will be compared to the total score from the MacCAT-T by using Pearson’s correlation test. The new instrument will then be piloted in real-time by street outreach clinicians to determine the acceptability and usefulness of the new instrument.

**Discussion:**

This research will build on the existing knowledge about assessing capacity to consent and will contribute new knowledge about assessing individuals whose judgment is impaired by substance use.

## Background

Substance misuse is associated with a high prevalence of physical and mental co-morbidities requiring medical care. In 2009 there were an estimated 4,049 hospitalizations as a result of illicit drug use in British Columbia (BC) Canada [1]. These data highlight the importance of providing prevention and treatment services to marginalized groups with addictions who are particularly vulnerable to intimidation, manipulation, coercion or exploitation [2]. This population often receives care in street outreach settings where clinician-client encounters are brief, creating complex challenges to clinicians who attempt to obtain consent for treatment to clients whose decision-making capacity may be impaired [3].

Informed consent for treatment is defined as an “individual’s autonomous and voluntary authorization of a medical decision” for which an individual has substantial understanding of the risks and benefits [2]. The BC Health Care (Consent) and Care Facility (Admission) Act [4] clearly stipulates that individuals must be capable of making rational decisions about their health and defines capacity as the ability to understand the medical care being offered (risks, benefits, and alternatives) and how it applies to the person being offered care [4]. This is contrasted with an equally important right to informed refusal. However, the practical methods for assessing capacity to consent (CTC) in public health care settings are unclear. Unlike the situation in most clinical settings, consent from clients in outreach settings cannot always be implied especially when the health professional seeks out clients rather than having them seek care. In these cases, verbal consent must be obtained and it is imperative that clinicians assess CTC before delivering health care.

### Assessing impaired CTC

Numerous tools have been developed to assess CTC for clinical treatment and/or research purposes. A recent systematic review [5] provides a comprehensive assessment of 23 instruments for assessing CTC. The majority of instruments have been developed for and validated in populations with mental illness such as depression, schizophrenia, and Alzheimer’s disease and involve a structured or semi-structured interview using hypothetical vignettes or real decision-making scenarios that last 15–90 minutes. These scales primarily focus on elements of understanding, recall of facts, the ability to appreciate the fact that the study or treatment involves them and the ability to state the reason for their decision. Current scales vary greatly in terms of standardization and scoring procedures.

The most widely used instrument is the MacArthur Competency Assessment Tool (MacCAT) (for treatment or for research) which has been shown to have good psychometric properties and takes 15–30 minutes to administer [6,7]. While these tools may be useful when the clinician has ample time to administer and score the instrument, it is impractical in street outreach settings, where clinical encounters are typically brief.

No tool, to our knowledge, has been developed to assess CTC among homeless individuals with problematic substance use. This group is unique in that some individuals may be better able to give consent when they are under the influence of a substance than when they are experiencing withdrawal symptoms. It is recognized that homeless adults with problematic substance use have a diminished CTC, making them vulnerable to exploitation [8]. Our research will address the particularly salient problem of assessing CTC in this population.

The purpose of this research study is to develop a brief tool that can be used by public health care clinicians to guide their assessment of their clients’ capacity to provide informed consent for medical care when under the influence of substances. This study has been approved by the University of British Columbia research ethics board.

### Research objectives

The objectives for this study are as follows: (1) to survey clinicians who provide services to clients who misuse substances to determine their current practices for assessing capacity to consent for clinical care, (2) to explore the experiences of providing consent from the perspective of people who misuse substances, and (3) to develop a brief assessment tool with acceptable psychometric properties that can be used to assess CTC for medical care in outreach settings.

## Methods/Design

A series of qualitative interviews will be conducted among a purposive sample of clinicians throughout BC who deliver care to homeless clients with problematic substance use. Recruitment will be conducted by email advertisement distributed by infectious control leaders in each health authority, and sampling will be done until theoretical saturation is reached (estimated to be 20 participants). Maximum-variation sampling will be employed, which allows exploration of both typical and unusual concepts across a broad range of settings [9]. Critical social theory and decision-making theory will be used to create a semi-structured interview guide to interview participants about their current practice for assessing CTC, i.e., the conceptual elements they consider when assessing capacity, the methods they use to assess these elements, and the threshold they use to make a final decision about capacity. Interviews will be conducted in a private office at the participant’s place of work.

A series of qualitative interviews will also be conducted with a convenience sample of adults throughout BC who are homeless and who self-report as abusing substances. Recruitment will be conducted through advertisement at community-based organizations and through word of mouth. Sampling will be done until theoretical saturation is reached (estimated to be 25 participants). The purpose of these interviews is to explore the experience of providing consent or obtaining medical care from the perspective of these individuals. Power inequities will also be explored. Interviews will be conducted in a private room where the community organization meets. A $20 cash honorarium will be provided to participants.

All interviews will be audiotaped and transcribed verbatim without personal identifiers. Concepts and themes will be identified in the data, and an interpretive description analysis [10], using both inductive (allowing concepts and themes to emerge from the data) and deductive (using concepts and themes that are described in the literature) approaches, will be conducted to determine how assessment of CTC can be translated into clinical practice.

### Instrument development

A comprehensive review of the literature will be conducted to examine the existing instruments for determining CTC. In addition, theoretical concepts related to CTC will be reviewed. The proposed tool will be constructed using theoretical concepts, legal concepts, and knowledge gained from qualitative interviews. Reliable, validated questions from existing instruments will be added to the list of possible questions.

The proposed questions will be provided to a panel of experts to establish item-objective congruency by rating each question (item) using the Osterlind method [11]. The panel of experts will include four doctors and four nurses from public health, one practitioner who delivers clinical care to the target population, one lawyer, one ethicist, an addictions specialist, and a psychiatrist. Experts will be encouraged to suggest other questions which may have been omitted. A modified Delphi process will be used to achieve group consensus about the relevancy of each question. Questions with discordant ratings will be further discussed in person until consensus is reached.

Once a near-final version of the instrument is created, pilot testing [12] will be conducted with five individuals from the target population. During this process, participants will be asked to respond to the questions and asked why they chose the response they chose and what the question meant to them. At this time, the number of response options will be assessed for appropriateness.

#### Instrument validation

Three hundred clients who are more than 18 years old, and who speak and read English, self-report as being homeless, and self-report abuse of substances will be recruited for a validation study through a recruitment poster placed in a downtown Vancouver community-based organization. A short questionnaire on demographics, substance use, and history of mental illness will be administered. Participants will be presented with a simulated consent for a hypothetical medical scenario. They will then be assessed by a psychiatrist to capture a clinical assessment of capacity. Next, participants will be administered the MacCAT for treatment (MacCat-T) tool followed by the administration of the new assessment tool, conducted by a research nurse blinded to the clinical and MacCAT-T result. A second researcher will observe the interview and score it independently and a Kappa statistic [13] will be calculated using SPSS 14 to determine inter-rater reliability. A Kappa score of >0.8 will be considered acceptable [14].

### Psychometric analysis

A combination of classical test theory and advanced psychometric methods will be used for the psychometric analysis. An item analysis will be conducted by examining the corrected item-total correlation to identify items that discriminate poorly [15,16]. Removal of items with corrected item-total correlation of <0.20 will be considered if the content is not considered clinically important. We aim to create an instrument that has the fewest items while maintaining good psychometric properties. The minimum number of items required to provide a reliability of at least 0.80 will be calculated using the Spearman Brown prophecy formula [17].

Guided exploratory factor analysis will be conducted on the final selection of items to confirm the assumptions for a Rasch model [18]. If multi-dimensionality is confirmed multidimensional Rasch analysis will be conducted. If unidimensionality is confirmed an unstandardized Cronbach Alpha will be calculated and a global summary score will be created. The global score and the MacCAT-T score will be compared using a Pearson’s correlation test. A cutoff value for the new instrument will be created by conducting a receiver operating characteristic analysis using a combination of the clinical assessment of capacity and the four domain scores from the MacCAT-T as the gold standard. If the clinical assessment and all four MacCAT-T domain scores indicate capacity, then the gold standard assessment will be recorded as “has capacity”. If any of these five elements indicates a lack of capacity, then the gold standard assessment will be recorded as “does not have capacity”. Results will be stratified by alcohol use only, drug use only, and mental status.

## Discussion

This study will address an important component of current practice for assessing CTC to treatment among populations with addictions who may have impaired CTC and thus be vulnerable to coercion. The new instrument will provide guidance to clinicians who deliver care to individuals with problematic substance use who may or may not be impaired during a clinical encounter, especially encounters that occur in outreach settings. No tools, to our knowledge, have been developed to assess CTC among these vulnerable individuals. Moreover, existing tools are cumbersome and may construct a barrier to care. While the study will be conducted in outreach settings, it is likely that this tool will be transferable to other settings where medical encounters with individuals impaired by substances are brief, such as emergency rooms, emergency medical services, and community clinics.

## Abbreviations

BC: British Columbia; CTC: Capacity to consent; MacCAT: MacArthur competency assessment tool; MacCAT-T: MacArthur competency assessment tool for treatment.

## Competing interests

All authors declare that they have no competing interests.

## Authors’ contributions

DT was responsible for drafting the article. DT, LM, JC, GO, and JAB were responsible for conception and design of the study. All authors were responsible for critical revision of important intellectual content and approval of the final version to be published.
